# Signal Subspace Smoothing Technique for Time Delay Estimation Using MUSIC Algorithm

**DOI:** 10.3390/s17122868

**Published:** 2017-12-10

**Authors:** Meng Sun, Yide Wang, Cédric Le Bastard, Jingjing Pan, Yuehua Ding

**Affiliations:** 1 Information Engineering College, Shanghai Maritime University, Shanghai 201306, China; msun@shmtu.edu.cn; 2 Institut d’Electronique et Télécommunications de Rennes, UMR CNRS 6164, Université de Nantes, 44306 Nantes CEDEX 3, France; yide.wang@univ-nantes.fr (Y.W.); cedric.lebastard@cerema.fr (C.L.B.); jingjing.pan1@etu.univ-nantes.fr (J.P.); 3 Cerema, Project-Team ENSUM, 49136 Les Ponts de Cé, France; 4 School of Electronic and Information Engineering, South China University of Technology, Wushan Road, Tianhe District, Guangzhou 510641, China

**Keywords:** Time Delay Estimation (TDE), signal subspace, sub-band averaging technique, radar

## Abstract

In civil engineering, Time Delay Estimation (TDE) is one of the most important tasks for the media structure and quality evaluation. In this paper, the MUSIC algorithm is applied to estimate the time delay. In practice, the backscattered echoes are highly correlated (even coherent). In order to apply the MUSIC algorithm, an adaptation of signal subspace smoothing is proposed to decorrelate the correlation between echoes. Unlike the conventional sub-band averaging techniques, we propose to directly use the signal subspace, which can take full advantage of the signal subspace and reduce the influence of noise. Moreover, the proposed method is adapted to deal with any radar pulse shape. The proposed method is tested on both numerical and experimental data. Both results show the effectiveness of the proposed method.

## 1. Introduction

Time Delay Estimation (TDE) has been a hot issue for many years. It has a great number of applications in radar, sonar, geophysics, medical imaging, communications, and so on. In [1], a unified approach to TDE from low-rate samples of the received signal is proposed for multipath channel estimation. A robust Capon beamformer based on time delay or time reversal is applied for ultrasound imaging in [2]. Moreover, the authors in [3] focus on TDE in both active and passive systems and take the envelope variation into account. In the field of civil engineering, time delays are important parameters for the quantitative interpretation of Ground-Penetrating Radar (GPR) data [4,5]. Within the range of centimeter wavelengths, GPR is often exploited for specific applications of stratified media, like roadways [6] or walls [7]. The structure (the layer thickness) of the media can be extracted from the time delays of the backscattered echoes associated with each interface and the dielectric constants of the various layers [6].

TDE is usually performed using the conventional FFT-based methods (inverse FFT or cross-correlation methods). However, the resolution of such methods is restricted by the frequency bandwidth of GPR. The case of small pavement thicknesses was studied in recent papers [8]. The main difficulty with data processing lies in the detection of close backscattered echoes. Some particular pavement materials are made up of thin layers (thickness ≤3 cm). The conventional methods are not able to distinguish close backscattered echoes (overlapped echoes). In this case, high resolution methods like MUSIC [9,10,11,12] and ESPRIT [13,14,15] are more suitable for TDE. Unlike the situations in [2,3], where the signals are supposed to be totally uncorrelated, in practice, the backscattered echoes are highly correlated (even coherent). Under this condition, the cross-correlation between the backscattered echoes may be too high to degrade the performance of the high resolution methods, such as the MUSIC algorithm, due to the rank loss of the data covariance matrix [16].

In order to apply high resolution methods, like in [1], for highly correlated echoes, sub-band averaging techniques are required to decorrelate the correlation between echoes. The well-known sub-band averaging technique, Spatial Smoothing Preprocessing (SSP), was firstly proposed in [17] and then developed in [18,19], which divides the whole frequency band into a series of overlapping sub-bands to obtain a new data covariance matrix with restored rank. Moreover, some improvements of SSP have been suggested in [14,15,20,21,22,23]. For example, [21] proposes an improved spatial smoothing technique, which takes full advantage of all the cross sub-band correlation matrices and auto-correlation matrices, while SSP only uses auto-correlation matrices.

However, the above preprocessing methods make use of the information from both the signal and noise subspaces of the data covariance matrix. In fact, it is not necessary to apply sub-band averaging techniques on the noise subspace. Furthermore, in this application, to apply the sub-band averaging technique, a whitening procedure is necessary. Due to the GPR pulse shape, the noise covariance matrix after the whitening procedure is no longer an identity matrix, which still contains the radar pulse. Therefore, in this paper, the MUSIC algorithm combined with an adaptation of the Signal Subspace Smoothing (SSS) technique [24] is proposed for TDE. The proposed method only makes use of the signal subspace and can be applied on any GPR pulse shape. The performance of the proposed method is tested on both numerical and experimental data. Both simulation and experimental results prove the effectiveness of the proposed method.

The rest of this paper is organized as follows: Section 2 gives the received radar data model. In Section 3, we present the proposed sub-band averaging technique. Simulation and experiment results are provided in Section 4. Finally, conclusions are drawn in Section 5.

Notations: ${\left(.\right)}^{T}$, ${\left(.\right)}^{*}$, ${\left(.\right)}^{-1}$ and ${\left(.\right)}^{H}$ denote the transpose, conjugate, inverse and conjugate transpose operations, respectively. E(:) denotes the ensemble average. diag(A) denotes the diagonal elements of matrix A. Vectors and matrices appear in boldface lower case letters and boldface capital letters, respectively.

## 2. Signal Model

In the roadway survey, we focus on the first layers, which are low-loss media. For pavement materials, the conductivity typically ranges within the interval $\left[10^{-3};10^{-2}\right]$ S/m, according to [25]. Thus, the media can be considered as a low-loss media. In addition, according to the work in [26], if the medium is slightly lossy, the dispersivity of the medium can be neglected. As a consequence, the echoes are simply time-shifted and attenuated copies of the transmitted signal [4,6,27,28]. Therefore, the received signal model can be written in the time domain as [8,14,22,29]:

(1) $$r\left(t\right)=\sum_{k=1}^{d}s_{k}e\left(t-t_{k}\right)+n\left(t\right)$$

For applying spectral analysis techniques to TDE, the received signal is usually formulated in the frequency domain. By using Fourier transform, the received signal model can be expressed as:

(2) $$r\left(f_{i}\right)=e\left(f_{i}\right)\sum_{k=1}^{d}s_{k}\exp\left(-j2\pi f_{i}t_{k}\right)+n\left(f_{i}\right)$$

where *d* is the number of backscattered echoes, which can be composed of multiple reflection echoes. *d* is either assumed to be known or estimated with some detection criteria [30]; $t_{k}$ is the time delay of the *k*-th echo; e(t) and $e(f_{i})$ are the radar pulse in the time and frequency domains, respectively; $s_{k}$ represents the amplitude of the *k*-th scattered echo; $n(f_{i})$ is an additive white Gaussian noise, with zero mean and variance $\sigma^{2}$; with $f_{i}=f_{1}+\left(i-1\right)\Delta f$, i=1,2…N the frequency, *N* the number of used frequencies, $f_{1}$ the lowest frequency of the studied frequency band and Δf the frequency step. Although the model is established in this work, it can also be applied in many applications such as radar, sonar, telecommunication, etc., which require the estimation of time delay or frequency. The received signal model can be written in the following vector form:

(3) $$\begin{array}{c} r=\Lambda As+n \end{array}$$

with the following notational definitions:

1. $r={\left[r\left(f_{1}\right)\;r\left(f_{2}\right)\;\cdots \;r\left(f_{N}\right)\right]}^{T}$ is the (N×1) received signal vector, called the observation vector, which may represent either the Fourier transform of the measured GPR signal or the measurements by a step-frequency radar;
2. $\Lambda =\mathrm{diag}\left(e\left(f_{1}\right),e\left(f_{2}\right),\ldots ,e\left(f_{N}\right)\right)$ is a (N×N) diagonal matrix, whose diagonal elements are the Fourier transform of the radar pulse e(t);
3. $A=\left[a\left(t_{1}\right)\;a\left(t_{2}\right)\;\ldots \;a\left(t_{d}\right)\right]$ is the (N×d) mode matrix;
4. $a\left(t_{k}\right)=[e^{-2j\pi f_{1}t_{k}}\;e^{-2j\pi f_{2}t_{k}}\;\ldots$$\;e^{-2j\pi f_{N}t_{k}}{]}^{T}$ is the mode vector;
5. $s={\left[s_{1}\;s_{2}\;\cdots \;s_{d}\right]}^{T}$ is a (d×1) vector;
6. $n={\left[n\left(f_{1}\right)\;n\left(f_{2}\right)\;\cdots \;n\left(f_{N}\right)\right]}^{T}$ is the (N×1) Gaussian noise vector with zero mean and covariance matrix $\sigma^{2}I$.

According to (3) and assuming the noise to be independent of the echoes, the covariance matrix Y of r can be written as:

(4) $$\begin{array}{c} \begin{array}{cc} Y & =E\left(rr^{H}\right)=\Lambda AE\left(ss^{H}\right)A^{H}\Lambda^{H}+E\left(nn^{H}\right)\\ & =\Lambda ASA^{H}\Lambda^{H}+\sigma^{2}I \end{array} \end{array}$$

where S is the (d×d)-dimensional covariance matrix of s; I is the identity matrix. To apply the sub-band averaging techniques, the influence of radar pulse must be removed from the radar signal. Therefore, in the following, the data are divided by the pulse, then the new observation vector $r^{\prime }$ can be written as $r^{\prime }=\Lambda^{-1}r=As+\Lambda^{-1}n=As+b$, with b the new noise vector. The new covariance matrix $R^{\prime }$ can be written as:

(5) $$R^{\prime }=E\left(r^{\prime }r^{\prime H}\right)=\Lambda^{-1}Y\Lambda^{-H}=ASA^{H}+\sigma^{2}\Sigma$$

with:

(6) $$\begin{array}{c} \Sigma =\Lambda^{-1}\Lambda^{-H}=\mathrm{diag}\left(\frac{1}{|e\left(f_{1}\right){|}^{2}},\frac{1}{|e\left(f_{2}\right){|}^{2}},\cdots ,\frac{1}{|e\left(f_{N}\right){|}^{2}}\right) \end{array}$$

## 3. Sub-Band Averaging Technique

In this section, we start with a short review of the conventional SSP and MSSP (Modified Spatial Smoothing Preprocessing) [22]. Then, the proposed SSS is presented.

### 3.1. Conventional SSP and MSSP

To mitigate the influence of the cross-correlation between the backscattered echoes, the conventional SSP/MSSP is applied on the data covariance matrix $R^{\prime }$. The whole frequency band with *N* sample points is partitioned into *M* overlapping sub-bands. Each sub-band is composed of *L* frequency points. Therefore, the maximum number of echoes that can be estimated is L−1. *N*, *M* and *L* are related to each other by N=L+M−1.

Let $r_{l}^{\prime }$ denote the (L×1) data vector on the *l*-th sub-band. It can be written as: $r_{l}^{\prime }=A_{1}D^{l-1}s+b_{l}$, where $b_{l}$ is the (L×1) noise vector on the *l*-th sub-band; $A_{1}$ denotes the first (L×d) sub-matrix of A ($A_{1}$ is independent of *l*); and D denotes the (d×d) diagonal matrix expressed as:

(7) $$D=\mathrm{diag}\left(e^{-2j\pi \Delta ft_{1}},\;\ldots \;,\;e^{-2j\pi \Delta ft_{d}}\right).$$

Therefore, the *l*-th sub-matrix of the covariance matrix $R^{\prime }$ can be written as follows:

(8) $$R_{l}=A_{1}D^{l-1}S{\left(D^{l-1}\right)}^{H}A_{1}^{H}+\sigma^{2}\Sigma_{l}$$

where $\Sigma_{l}$ is the *l*-th sub-matrix of the noise matrix Σ.

According to SSP [22], the rank restored covariance matrix $R_{\mathrm{SSP}}$ can be expressed as follows:

(9) $$\begin{array}{cc} R_{\mathrm{SSP}} & =\frac{1}{M}\sum_{l=1}^{M}R_{l}. \end{array}$$

Likewise, in MSSP [22], the modified covariance matrix $R_{\mathrm{MSSP}}$ can be written as follows:

(10) $$\begin{array}{cc} R_{\mathrm{MSSP}} & =\frac{1}{2M}\sum_{l=1}^{M}\left\{R_{l}+JR_{l}^{*}J\right\} \end{array}$$

where J is the (L×L) exchange matrix. From (9) and (10), the conventional sub-band averaging techniques are directly applied on the data covariance matrix.

### 3.2. Proposed SSS Technique

In this paper, the SSS technique is introduced, which is applied only on the signal subspace. It suffers the rank loss, but contains all the information of the backscattered echoes. Therefore, in the first step, we need to apply the EigenValue Decomposition (EVD) on the covariance matrix Y:

(11) $$\begin{array}{c} Y=U_{s}\Lambda_{s}U_{s}^{H}+U_{n}\Lambda_{n}U_{n}^{H} \end{array}$$

where $\Lambda_{s}$ is a ($d^{\prime }\times d^{\prime }$) diagonal matrix containing larger eigenvalues. For totally correlated echoes, the number of larger eigenvalues $d^{\prime }$ is 1, $d^{\prime }\leq d$. The corresponding eigenvectors are in the $\left(N\times d^{\prime }\right)$ matrix $U_{s}$. $\Lambda_{n}$ is a ($\left(N-d^{\prime }\right)\times \left(N-d^{\prime }\right)$) diagonal matrix, which contains the other smaller eigenvalues, and the associated eigenvectors are in the $\left(N\times \left(N-d^{\prime }\right)\right)$ matrix $U_{n}$. According to [9], there exists a $\left(d\times d^{\prime }\right)$-dimensional full rank matrix T, with $U_{s}=\Lambda \mathrm{AT}$. The signal subspace can be expressed as:

(12) $$\begin{array}{c} Y_{s}=U_{s}\Lambda_{s}U_{s}^{H}=\Lambda AT\Lambda_{s}T^{H}A^{H}\Lambda^{H}. \end{array}$$

Dividing matrix $Y_{s}$ by the radar pulse, we have the new signal subspace matrix $R_{s}$ as:

(13) $$\begin{array}{c} R_{s}=\Lambda^{-1}Y_{s}\Lambda^{-H}=AT\Lambda_{s}T^{H}A^{H}. \end{array}$$

The *l*-th (L×L) sub-matrix of $R_{s}$ is written as $R_{sl}$. Then, we apply the principle of the MSSP technique on $R_{s}$. Accordingly, the rank restored covariance matrix R can be written as the average of *M* overlapping sub-matrices as follows:

(14) $$\begin{array}{c} R=\frac{1}{2M}\sum_{l=1}^{M}\left\{R_{sl}+JR_{sl}^{*}J\right\} \end{array}$$

After decorrelation, subspace methods, like the MUSIC algorithm [31], can be applied for TDE. In addition, the amplitudes of echoes can be calculated by the least squares method [5], and then, the relative permittivity of each layer can be estimated. Finally, the layer thickness can be estimated using the estimated time delay and relative permittivity. Compared with conventional approaches, the proposed method makes full use of signal subspace and is more robust to the noise impact. Moreover, the proposed method is able to be applied on any shape of radar pulse.

## 4. Simulations and Experiment

### 4.1. Simulation Results

**Figure 1:**
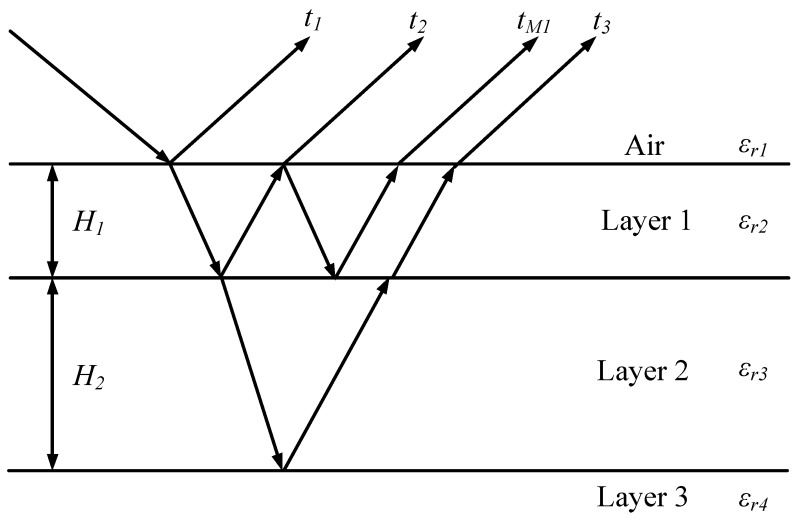
Pavement configuration; $t_{k}$ represents the time delay of the *k*-th interface, and $t_{M1}$ represents the time delay of a multiple echo.

In this section, the performance of the proposed method is tested on simulated data obtained from (3). In the simulation, the media are composed of three homogeneous layers. The pavement configuration is shown in Figure 1. Assume the observation is performed at nadir and far-field. The parameters of the media are chosen as follows: the relative permittivities of the three layers are $\varepsilon_{r2}=4$, $\varepsilon_{r3}=8$ and $\varepsilon_{r4}=9$, respectively. The thicknesses of Layer 1 and Layer 2 are $H_{1}$ and $H_{2}$, respectively. The thickness of Layer 3 is assumed as infinite. The frequency band used is [0.5–2.5] GHz, with a 0.05-GHz frequency step (41 frequency samples). The number of sub-bands (*M*) is equal to 20. The data covariance matrix is estimated from 500 independent snapshots. The Signal-to-Noise Ratio (SNR) is defined as the ratio between the power of the last primary echo and the noise variance.

In the first simulation, the pseudo-spectrum of the proposed method is calculated and compared with that of MUSIC-MSSP. SNR is fixed at 10 dB. Two cases are studied with different thicknesses:

- Case a. $H_{1}=30$ mm and $H_{2}=60$ mm: The received signal is made up of three primary echoes and one multiple echo (one multiple reflection inside the first layer is considered). The corresponding time delays ($t_{1},\;t_{2},\;t_{M1},\;t_{3}$) are 1.00 ns, 1.40 ns, 1.80 ns and 2.53 ns, respectively. The first three echoes are overlapped.
- Case b. $H_{1}=20$ mm and $H_{2}=10$ mm: The received signal is made up of three primary echoes. The corresponding time delays ($t_{1},\;t_{2},\;t_{3}$) are 1.00 ns, 1.27 ns and 1.46 ns, respectively. The three echoes are overlapped.

**Figure 2:**
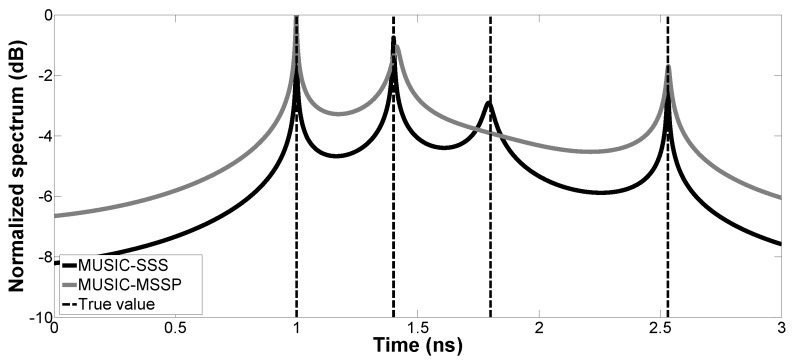
Case a, pseudo-spectrum of MUSIC-SSS and MUSIC-MSSP for TDE with SNR =10 dB.

**Figure 3:**
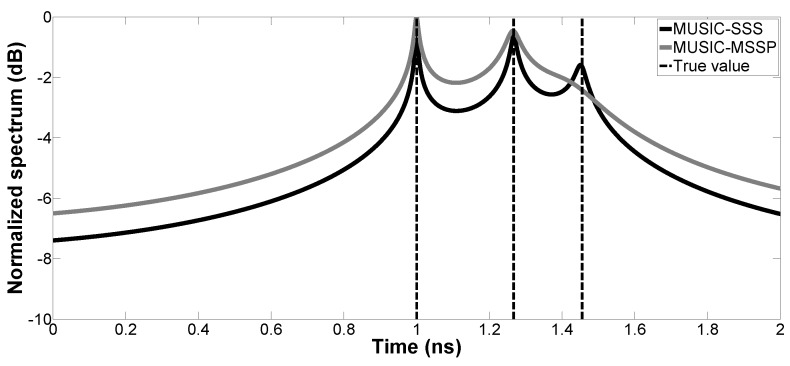
Case b, pseudo-spectrum of MUSIC-SSS and MUSIC-MSSP for TDE with SNR =10 dB.

The pseudo-spectrums of the proposed method and MUSIC-MSSP are presented in Figure 2 and Figure 3. The time delays of the backscattered echoes are well estimated in Cases a and b by the proposed method. The peaks of MUSIC-SSS correspond to the true time delays, thanks to its high resolution and robustness to the noise impact. However, in Case a, MUSIC-MSSP fails to estimate the weak multiple reflection echo; in Case b, MUSIC-MSSP cannot estimate the third primary echo, as the third echo is too close to the second echo.

In the second simulation, the performance of the proposed method versus SNR is evaluated by a Monte Carlo process with 200 independent runs. The Root-Mean-Squared Error (RMSE) of the studied parameter is defined as:

(15) $$\begin{array}{c} RMSE=\sqrt{\frac{1}{U}\sum_{u=1}^{U}{\left({\hat{z}}_{u}-z\right)}^{2}} \end{array}$$

where ${\hat{z}}_{u}$ denotes the estimated parameter for the *u*-th run of the algorithm and *z* can be the true value of the *k*-th time delay ($t_{k}$) or layer thickness ($H_{1}$ or $H_{2}$); *U* is the number of independent runs. SNR varies from 0–30 dB. Only Case a is studied. The proposed method is compared with MUSIC-MSSP.

**Figure 4:**
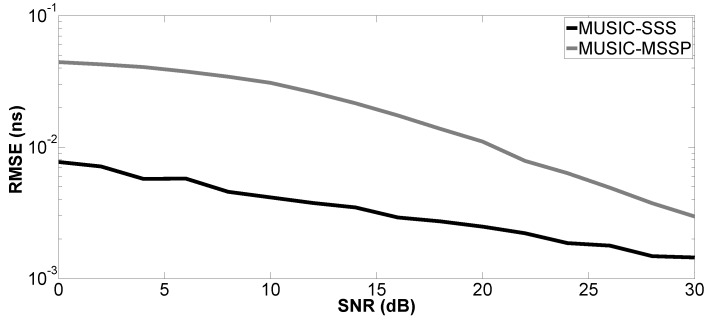
RMSE on the estimated time delay ${\hat{t}}_{1}$ versus SNR.

**Figure 5:**
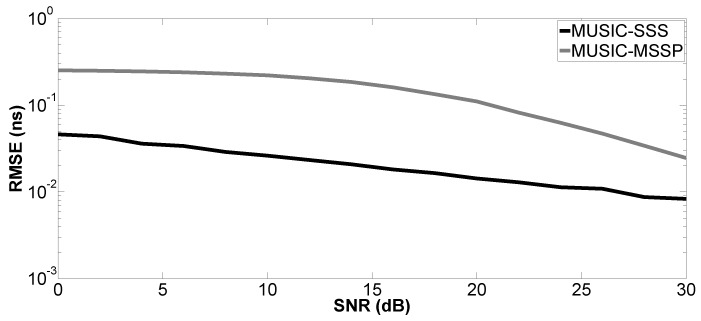
RMSE on the estimated time delay ${\hat{t}}_{2}$ versus SNR.

**Figure 6:**
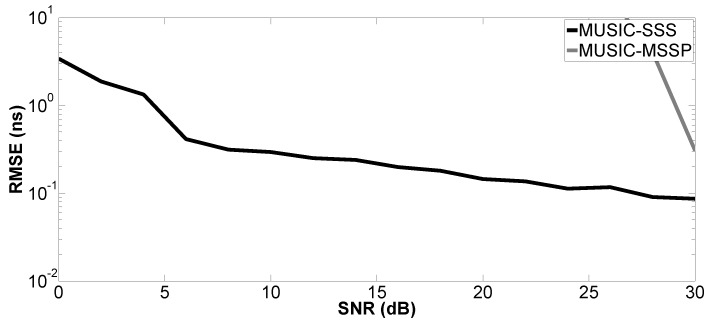
RMSE on the estimated time delay ${\hat{t}}_{M1}$ versus SNR.

**Figure 7:**
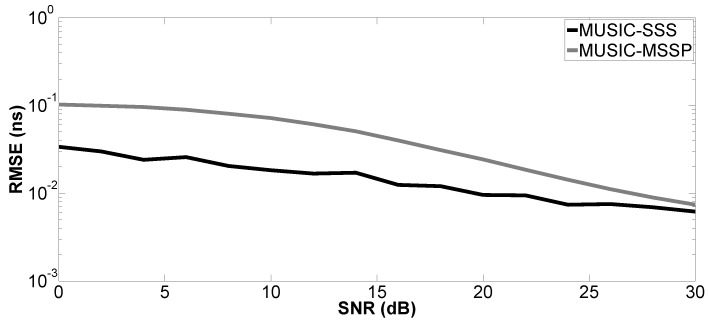
RMSE on the estimated time delay ${\hat{t}}_{3}$ versus SNR.

**Figure 8:**
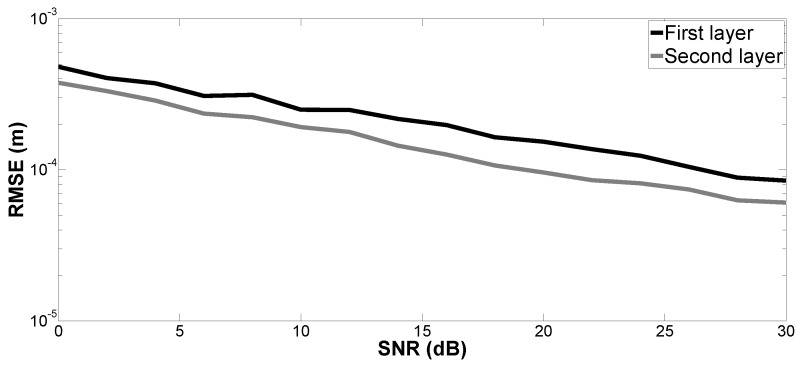
RMSE on the estimated thicknesses of the first and second layers: $H_{1}$ and $H_{2}$ versus SNR.

Figure 4, Figure 5, Figure 6 and Figure 7 show the RMSE on the estimated time delays by the proposed method and MUSIC-MSSP. The RMSE on the estimated time delays continuously decreases with increasing SNR for both compared methods. The proposed method has a more significant decrease of the RMSE than that of MUSIC-MSSP; MUSIC-MSSP fails to estimate the multiple echo at low SNR, as shown in Figure 6. Moreover, the RMSE also depends on the amplitude of the backscattered echo. The echo with the larger amplitude has smaller RMSE on TDE. Besides, the RMSE on ${\hat{t}}_{3}$ is smaller than that of the other three time delays because this echo is not overlapped with the others. It is clear that the RMSE obtained by the proposed method is smaller than that obtained by MUSIC-MSSP for all the considered values of SNR, which means that the proposed method has better accuracy and outperforms MUSIC-MSSP. Furthermore, Figure 8 shows the RMSE on the estimated thicknesses of the first and second layers by the proposed method. It estimates the layer thickness with small RMSE and gives relatively good performance in thickness estimation.

**Figure 9:**
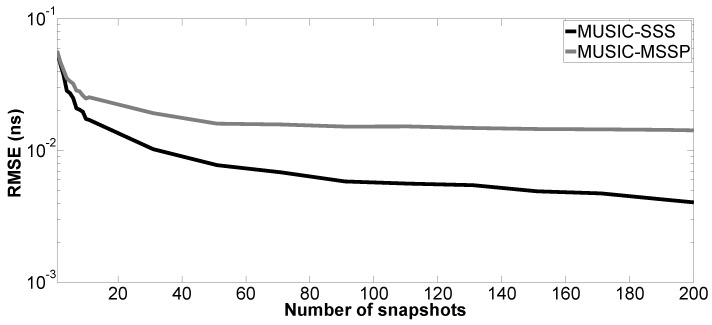
RMSE on the estimated time delay ${\hat{t}}_{1}$ versus the number of snapshots.

**Figure 10:**
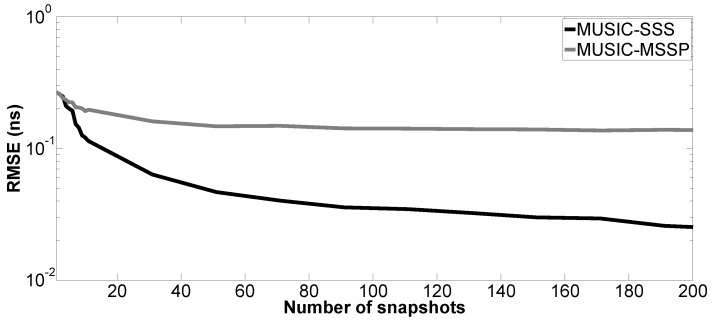
RMSE on the estimated time delay ${\hat{t}}_{2}$ versus the number of snapshots.

**Figure 11:**
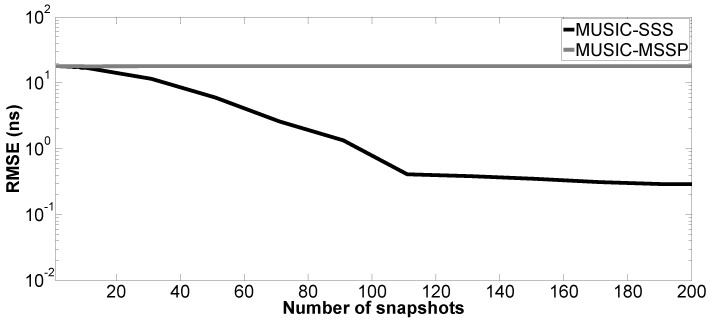
RMSE on the estimated time delay ${\hat{t}}_{M1}$ versus the number of snapshots.

**Figure 12:**
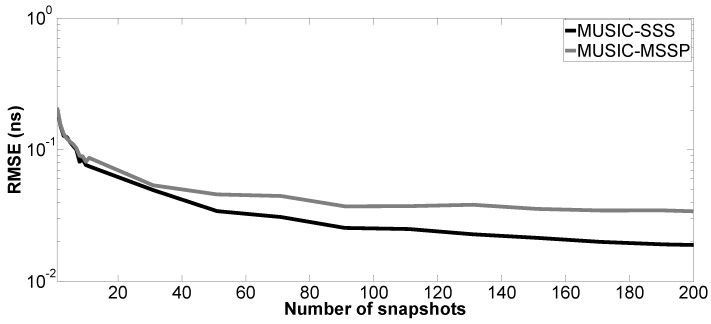
RMSE on the estimated time delay ${\hat{t}}_{3}$ versus the number of snapshots.

Moreover, the performance of the proposed method versus the number of snapshots is tested. The number of Monte Carlo processes is equal to 200. The parameter setting in Case a is applied here; SNR is fixed at 10 dB. Figure 9, Figure 10, Figure 11 and Figure 12 show the RMSE of the proposed method and MUSIC-MSSP as a function of the number of snapshots. Similar to the second simulation, the RMSE on the estimated time delays using the proposed method continuously decreases as the number of snapshots increases. In addition, it can be seen in Figure 11 that MUSIC-MSSP fails to estimate the multiple echo for each number of snapshots. Finally, it can be concluded that the proposed method has better accuracy than MUSIC-MSSP.

### 4.2. Experimental Results

**Figure 13:**
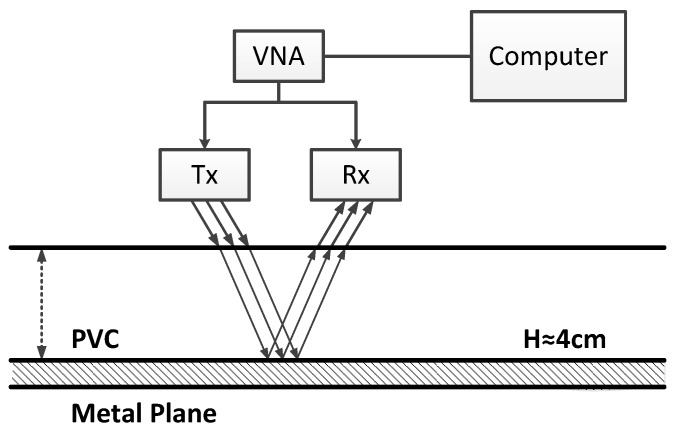
Experimental setup.

**Figure 14:**
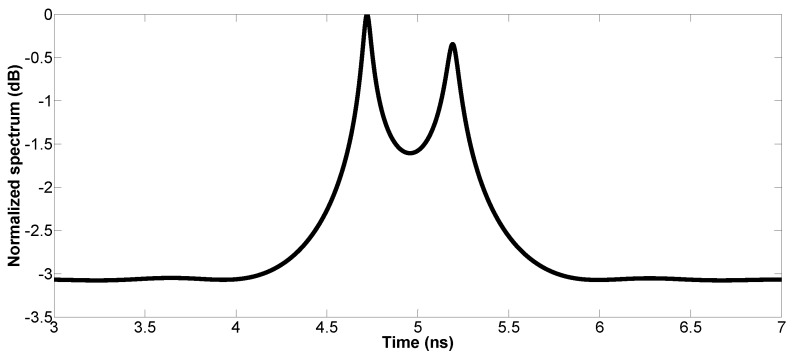
Pseudo-spectrum of MUSIC using the SSS technique with experimental data.

In addition, the proposed method is tested on experimental data. A mono-static step frequency radar is used, which is composed of a Vector Network Analyzer (VNA) and a bistatic antenna device whose Transmitter (Tx) and Receiver (Rx) are close to each other. The antennas are about 70 cm above the tested media, which allows them to be in the far-field condition. As shown in Figure 13, the tested media are comprised of a PolyVinyl Chloride (PVC) slab set on a metal plane. The thickness of the PVC is about 4 cm, and its relative permittivity is $\varepsilon_{r}=2.97+0.0015j$. The radar frequency bandwidth ranges from 1.0 GHz–2.6 GHz, with a 0.02-GHz frequency step (81 frequency samples). The radar pulse is measured with a metal plane [8]. In the experiment, the proposed method is tested with the number of sub-bands equal to nine. Figure 14 shows the experimental result of the proposed method. The estimated time delay between two echoes is 0.47 ns, and the estimated thickness of the PVC is approximately 4.09 cm (the relative error is 2.25%).

## 5. Conclusions

In this paper, we propose a MUSIC algorithm combined with the proposed SSS technique for TDE. Compared with conventional methods, the proposed method has three major merits: firstly, it takes full advantage of the signal subspace; secondly, it is robust to the influence of noise; thirdly, it can be applied to any radar pulse. The performance of the proposed method is compared with the conventional MSSP. Both numerical and experimental results show the stability and robustness of the proposed method in TDE.

## Abbreviations

The following abbreviations are used in this manuscript:

TDE	Time Delay Estimation
GPR	Ground-Penetrating Radar
FFT	Fast Fourier Transform
MUSIC	Multiple Signal Classification
ESPRIT	Estimation of Signal Parameters via Rational Invariance Technique
SSP	Spatial Smoothing Preprocessing
MSSP	Modified Spatial Smoothing Preprocessing
SSS	Signal Subspace Smoothing
EVD	EigenValue Decomposition
SNR	Signal-to-Noise Ratio
RMSE	Root Mean Square Error
VNA	Vector Network Analyzer
PVC	PolyVinyl Chloride
